# A Theoretical Study of the Relationship between the Electrophilicity ω Index and Hammett Constant σ_p_ in [3+2] Cycloaddition Reactions of Aryl Azide/Alkyne Derivatives

**DOI:** 10.3390/molecules21111434

**Published:** 2016-10-27

**Authors:** Hicham Ben El Ayouchia, Hafid Anane, Moulay Lahcen El Idrissi Moubtassim, Luis R. Domingo, Miguel Julve, Salah-Eddine Stiriba

**Affiliations:** 1 Laboratoire de Chimie Analytique et Moléculaire, Faculté Polydisciplinaire de Safi, Université Cadi Ayyad, Safi 46010, Morocco; belayou@gmail.com (H.B.E.A.); ananehafid@gmail.com (H.A.); ml_elidrissi@yahoo.fr (M.L.E.I.M.); 2 Departamento de Química Orgánica, Universidad de Valencia, Avda. Dr. Moliner 50, Burjassot 46100, Valencia, Spain; domingo@utopia.uv.es; 3 Instituto de Ciencia Molecular/ICMol, Universidad de Valencia, C/Catedrático José Beltrán No. 2, Paterna 46980, Valencia, Spain; miguel.julve@uv.es

**Keywords:** [2+3] cycloaddition reactions, arylazides, arylalkynes, substituent effects, electrophilicity index, Hammett constants

## Abstract

The relationship between the electrophilicity ω index and the Hammett constant σ_p_ has been studied for the [2+3] cycloaddition reactions of a series of *para*-substituted phenyl azides towards *para*-substituted phenyl alkynes. The electrophilicity ω index—a reactivity density functional theory (DFT) descriptor evaluated at the ground state of the molecules—shows a good linear relationship with the Hammett substituent constants σ_p_. The theoretical scale of reactivity correctly explains the electrophilic activation/deactivation effects promoted by electron-withdrawing and electron-releasing substituents in both azide and alkyne components.

## 1. Introduction

**Scheme 1:**
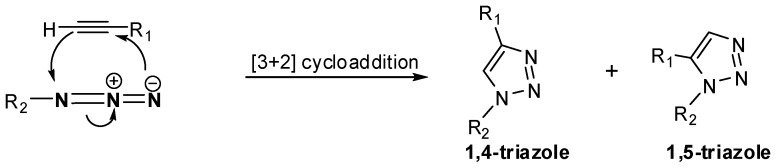
Mechanism of the Huisgen azide–alkyne 32CA reaction.

The [3+2] cycloaddition (32CA) reaction between azides, acting as the three-atom-component (TAC) and carbon–carbon triple bonds is a classical organic reaction initially established by Huisgen, in which five-membered 1,2,3-triazolic compounds are prepared as a mixture of 1,4 and 1,5-regioisomers. Since then, triazoles have played central roles in coordination chemistry as nitrogen-containing heterocyclic ligands, in bioconjugation issues, and in peptide-based drug design by mimicking peptide and disulfide bonds, leading to secondary structural components of peptides [1,2,3]. The interest in triazoles has recently attracted great attention, since the introduction of copper(I)-catalyzed 32CA reactions between azides and alkynes (CuCAA) [4]. The CuCAA reaction is classified as a click chemical process that takes place in a regioselective manner, giving only the 1,4-disubstituted triazole isomer [5]. A large number of mechanistic investigations were established describing the mechanistic pathways of these 32CA reactions (Scheme 1) [6,7].

**Scheme 2:**
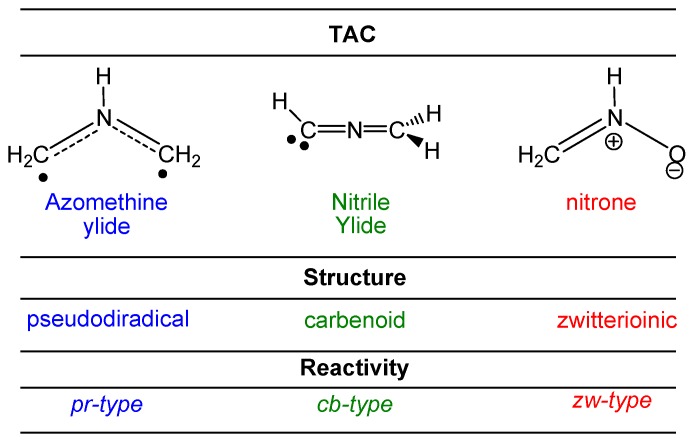
Electronic structure of three-atom-components (TACs) and the proposed reactivity types in [3+2] cycloaddition (32CA) reactions.

Unlike 1,3-dienes participating in Diels–Alder reactions [8], the electronic structure of TACs participating in 32CA reactions strongly depends on the type and hybridization of the atoms present in the TAC. Thus, depending on their electronic structure, TACs have recently been classified as *pseudodiradical*, carbenoid, and zwitterionic TACs (see Scheme 2) [9,10]. It should be noted that only 1,2-zwitterionic TACs such as nitrone or azides have the electronic structure of a 1,3-dipole as Huisgen proposed [6].

Molecular Electron Density Theory [10,11] studies devoted to the understanding of the mechanisms of 32CA reactions have allowed the establishment of a useful classification of these reactions into *pseudodiradical*-type (*pr-type*) [9], carbenoid-type (*cb-type*) [10], and zwitterionic-type (*zw-type*) [9] reactions, in such a manner that TACs with a *pseudodiradical* character participate in *pr-type* 32CA reactions taking place easily through earlier transition states (TSs) with a very low polar character [9,12]; TACs with a carbenoid character participate in *cb-type* 32CA reactions whose feasibility depends on the polar character of the reaction (i.e., the nucleophilic character of the carbenoid TAC and the electrophilic character of the ethylene derivative) [10]; and finally, TACs with a zwitterionic character participate in *zw-type* 32CA reactions controlled by nucleophilic/electrophilic interactions taking place at the TSs, similarly to *cb-type* reactions [9,13].

The simplest azide, HNNN—which has a zwitterionic structure—presents low nucleophilic character, *N* = 1.81 eV, and very low electrophilic character, ω = 0.66 eV [13]. Consequently, it is expected that it participates neither as nucleophile nor as electrophile in *zw-type* 32CA reactions [13]. Consequently, the simplest azide must be electronically activated in order to easily participate in a *zw-type* 32CA reaction with low activation energy.

Substituent effects on the TACs (regarded as a zwitterionic species), and alkynes’ reactivities in the 32CA reaction rate and stereoselectivity leading to triazoles remain unfortunately unclear, and need further theoretical investigation [14]. A very recent report shows that the substituent effect could change the reaction mechanism from concerted single-step to stepwise pathway for some of the TAC azide compounds [15]. The linear free energy relationship is among the empirical methods that can help to develop an understanding of the substituent effects on the reactivity of both azides and alkynes. Linear free-energy relationships are empirical relationships between thermodynamic quantities known as extra-thermodynamic equations [16]. They have been invaluable in the investigation of the structural properties and reactivities of organic compounds in solution. Among them, the Hammett constants σ_p_ have been permanently used in relating the nature of the substituents to their electronic effects (inductive and resonance) on chemical reactivity and other properties [17]. Today, these constants remain an excellent guide for structure–property and structure–activity studies [18]. The fact that the constants derived from these equations were empirical led to efforts to look for correlations between them and other theoretically-derived parameters which might be obtained from quantum chemical calculations. Several indices derived from conceptual density functional theory (DFT) have been increasingly used in the interpretation of organic reactivity. They include reactivity indices like chemical potential, hardness, softness, electrophilicity, and nucleophilicity indices [19].

It is known that the Hammett equation [2,3] relates the relative magnitude of the equilibrium constants to a reaction constant (ρ) and a substituent constant (σ_p_), according to Equation (1) [20].

log(K/K_0_) = ρ σ_p_ (1)

From a theoretical point of view, the electrophilic and nucleophilic behaviors of organic molecules can be characterized by using the reactivity indices defined within the conceptual DFT framework [19,21]. Parr et al. [22] introduced the following definition of the electrophilicity ω index of a molecule in terms of its chemical potential μ and chemical hardness η through Equation (2).

⍵ = μ^2^/2η (2)

where μ and η are the electronic chemical potential and chemical hardness of the ground state of the atoms and molecules, respectively [23]. Electrophilicity index ω measures the stabilization in energy when the system acquires an additional electronic charge from the environment [22]. By definition, it encompasses both the ability of an electrophile to acquire additional electronic charge and the resistance of the system to exchanging electronic charge with the environment.

Linear relationships between σ_p_ and ω have recently been obtained for *para*-substituted benzyl cations. Domingo et al. have systematically compared the experimental σ_p_ values and electronic electrophilicity index ω for a series of forty-two substituted ethylene derivatives [24]. They developed a statistical procedure to obtain intrinsic electronic contributions to σ_p_ based on the comparison between the experimental Hammett constant σ_p_ and the electrophilicity index ω, evaluated for a series of functional groups that are present in organic compounds.

**Figure 1:**
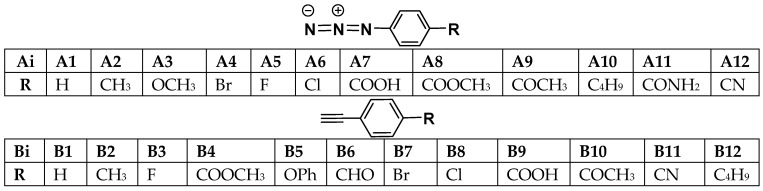
General structures of the (**A**) azide and (**B**) alkyne derivatives used in the 32CA reactions studied in this work.

Herein, we present a theoretical model to quantitatively describe the Hammett substituent constants σ_p_ in terms of the global electrophilicity ω of azides and alkynes used in 32CA reactions by using a global electrophilicity index ω as well as the logarithm of the global electrophilicity ratios of *para*-substituted and unsubstituted compounds (see Figure 1). The global electrophilicity index ω of a series of aromatic azides and alkynes is classified within an absolute scale in order to illustrate the rationalization of the substituent effects on the electrophilic activation/deactivation of the substrates. Indeed, such aromatic substrates were chosen instead of aliphatic substrates because of the substantial electronic effect of the *para*-substituted group on either azide or carbon–carbon triple bond facilitated by the electronic transmission through the aromatic π-conjugated system.

## 2. Results and Discussion

**Figure 2:**
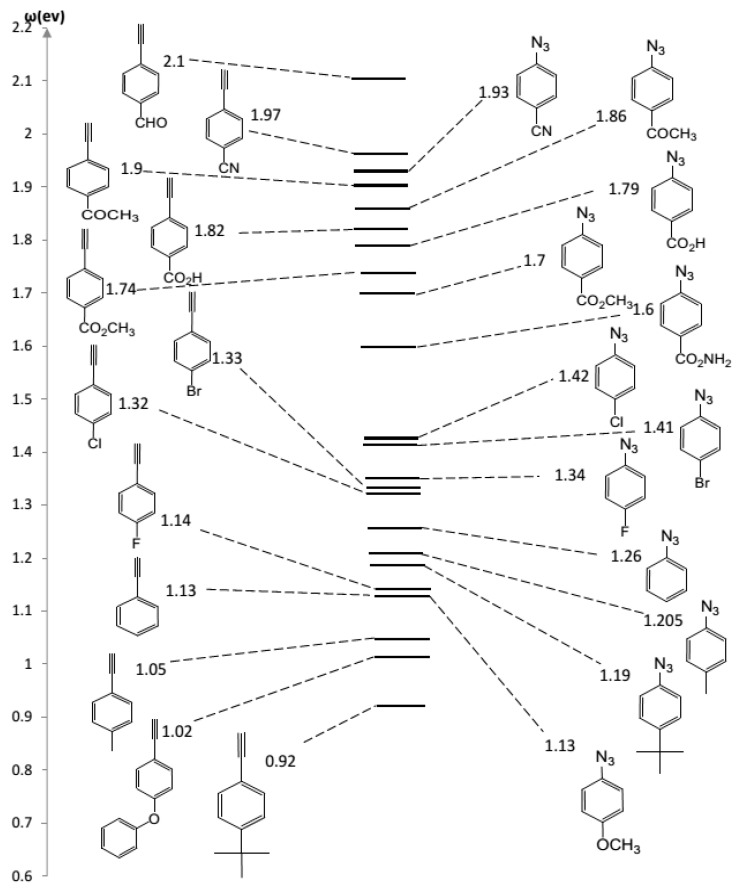
Theoretical scale of global electrophilicity ω for substituted alkynes and azides studied in 32CA reactions.

The global electrophilicity patterns of the substituted azide (**A**) and alkyne (**B**) derivatives used in the 32CA reactions are ranked in Figure 2. It can be seen that compounds with electron-withdrawing (EW) substituents occupy the top of the scale, while compounds with electron-releasing (ER) substituents are in the bottom. Alkynes display slightly higher electrophilicity values than azides with similar EW substituents, and it is a little lower when using ER ones. It is also possible to rationalize the electrophilic activating/deactivating effects promoted by substituent groups in both azide and alkyne compounds. For instance, the unsubstituted reference TAC **A1**(–H) has an electrophilic value of (ω = 1.26 eV). Its *para*-substitution by the weak ER methyl (–CH_3_) group results in an electrophilic deactivation as shown in compound **A2** (ω = 1.20 eV).

By substitution of the same *para* position with the stronger ER methoxy (–OCH_3_) group, an even higher electrophilic deactivation is observed in compound **A3** (ω = 1.13 eV). As expected, the substitutions with EW groups show electrophilic activation. For example, substitution with fluorine causes an activation of about 0.09 eV in compound **A5** with respect to the unsubstituted compound **A1**. Whereas the most efficient activation with respect to compound **A1** is achieved by the cyano group (–CN) as found in compound **A12** (ω = 1.93 eV).

For the series of alkynes, a similar picture is obtained. So, starting from the reference compound **B1** (ω = 1.13 eV), the *para* substitution with fluorine (–F), chlorine (–Cl), and bromine (–Br) atoms results in an electrophilic activation in compounds **B3** (ω = 1.14 eV), **B7** (ω = 1.32 eV), and **B8** (ω = 1.33 eV). The highest activation effect is achieved by EW carbonyl (–CHO) substitution, in compound **B6** (ω = 2.10 eV). In this series, the electrophilic deactivation was caused by ER groups as found, for example, with the methyl substituting –CH_3_ in compound **B2** (ω = 1.05 eV). In line with that, the substitution by a stronger ER group such as (–*t-*Bu) results in a higher electrophilic deactivation, as found for compound **B12** (ω = 0.92 eV).

As in single-substituted molecules, a low electrophilicity index ω has been correlated with good nucleophiles [25]. It is expected that the more favorable azide/alkyne *zw-type* 32CA reactions take place when both regents are located at the extreme of Figure 2 (i.e., good electrophilic azides with good nucleophilic alkyne, or vice versa).

The global values of electrophilicity indexes ω for both azides and alkynes series for the ground state of the substituting agents as well as the Hammett substituent constants σ_p_ are listed in Table 1. The utility of a reactivity scale has been clearly illustrated by Mayr et al. [26,27]. Such a reactivity scale should be able to address fundamental questions concerning reaction feasibility, intramolecular selectivity, and other important reactivity aspects.

**Figure 3:**
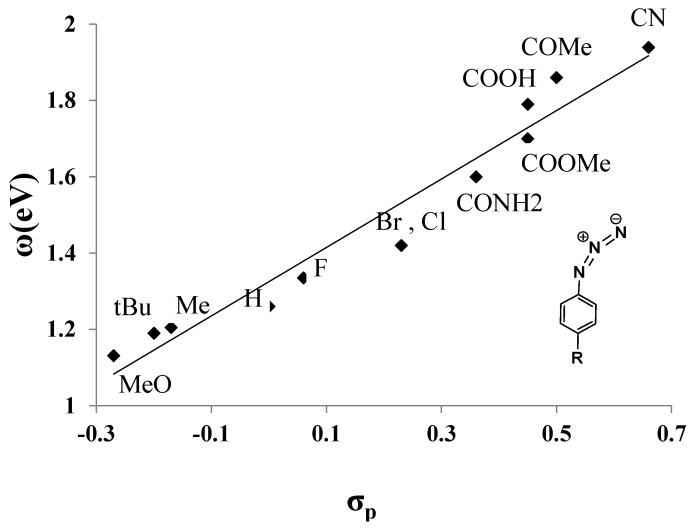
Plot of the global electrophilicity ω versus the Hammett constants for *para* substituents in the azide series. The value of the regression coefficient for the least-squares fit to a linear plot is *R*^2^ = 0.96.

**Figure 4:**
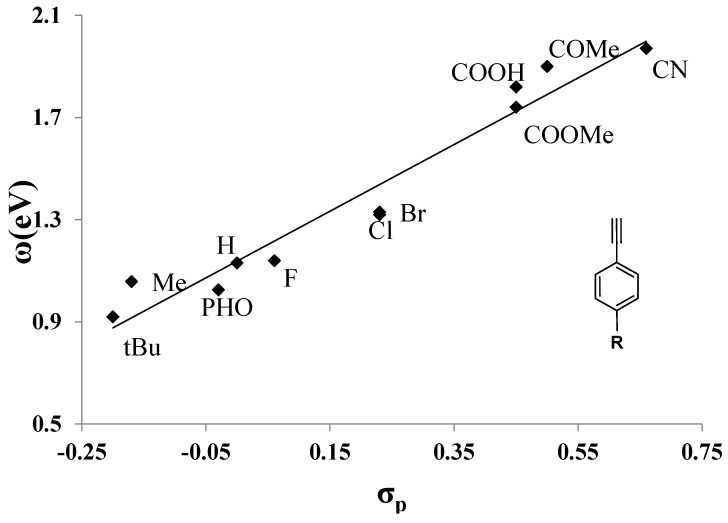
Plot of the global ω electrophilicity *against the* Hammett constants for *para* substituents in the alkyne series. The value of the regression coefficient for the least-squares fit to a linear plot is *R*^2^ = 0.95.

Figure 3 and Figure 4 show a positive slope in the relationship between the Hammett constants σ_p_ of *para*-substituents in the azide/alkyne derivatives and the global electrophilicity index ω. It should be noted that the *para* position is the best one that leads to the activation of the azide and carbon–carbon triple bond groups and then to a nice correlation ω = *f*(σ_p_), by comparison with the *ortho-* and *meta-*positions.

The resulting regression equations of the logarithm of the global electrophilicity ratio (ω/ω_H_) versus the Hammett substituent constants σ_p_ of the substituted and unsubstituted molecules at the ground state for the same series of azides and alkynes, evaluated at the DFT/6-31G(d) level are also represented in Figures S1 and S2 (Supplementary Materials), obeying Equations (3) and (4), respectively.

Log (ω/ω_H_) = 0.26 σ_p_ + 0.02 (3)

Log (ω/ω_H_) = 0.40 σ_p_ − 0.01 (4)

The two plots represented in Figure 2 and Figure 3, as well as those represented in Figures S1 and S2, confirm the existence of good linear relationships between both variable parameters, namely the global electrophilicity ω descriptor of reactivity and the Hammett substituent constants σ_p_ of the series of azides and alkynes involved in 32CA reactions. However, some improvements can still be made by taking into account the catalyst used, particularly under the click regime of 32CA reactions, and evaluating the global electrophilicity of both substrates at a more realistic stage of the reaction—namely, the transition state (TS). The electrophilicity scale correctly accounts for the electrophilic activation/deactivation effects promoted by the substituents in the ground state of the electrophiles involved in 32CA reactions. Indeed, it is proven that the EW substituents increase the global electrophilicity of the reagent (either azide or alkyne), unlike ER substituents, which behave oppositely. This behavior is due to the great activation of the azide, and alkyne’s carbon–carbon triple bond function promoted by electronic resonance effects of the EW substituents, instead of the great stabilization and then deactivation promoted by the ER groups in the alkyne and azide derivatives. In light of the above-mentioned results, it appears that the 32CA reaction of a 4-substitued phenyl azide with an activating EW substituent to a 4-substituted phenyl alkyne with a deactivating ER group is more favorable, and may lead to a fast and quantitative reaction, and vice-versa.

## 3. Computational Details

All chemical structures discussed in this study are depicted in Figure 1. They were optimized at the B3LYP/6-31G(d) level of theory using the Gaussian 09 suite of programs [28].

The global electrophilicity index, ω [22], which measures the stabilization in energy when the system acquires an additional electronic charge ∆*N* for the environment, is given by the following expression, ⍵ = μ^2^/2η in terms of the electronic chemical potential (μ) and the chemical hardness (η). Both quantities may be approached in terms of the one-electron energies of the frontier molecular orbital HOMO and LUMO, and ε_H_ and ε_L_ as μ = (ε_H_ + ε_L_)/2 and η = (ε_L_ − ε_H_) respectively [23]. Although absolute values of the reactivity indices can change with the computational level, functionals, and bass sets, the relative position of the compounds in the corresponding scales does not modify. So, we have selected the B3LYP/6-31G(d) level used in most of the scales of the reactivity indices [14,29].

## 4. Conclusions

In conclusion, the linear correlation between the global electrophilicity indices ω and its logarithm for a series of *para*-substituted phenyl azide and *para*-substituted phenyl alkyne compounds participating in 32CA reactions exhibit a high correlation coefficient with the experimental Hammett substituent constants σ_p_. The reactivity of both azide and alkyne derivatives is promoted by the electronic withdrawing and releasing effects of the substituents, leading to their stabilization or destabilization. A theoretical scale of the global electrophilicity ω of the two series of azides and alkynes considered in this study by using the global electrophilicity index ω is nicely described for the first time.

## Figures and Tables

**Table 1:** Electronic chemical potential (μ), chemical hardness (η), global electrophilicity (ω), in eV, Hammett constants σ_p_^a^, and the logarithm of the global electrophilicity ratio (ω/ω_H_).

Compound	R	μ	η	ω	σ_p_^a^	Log(ω/ω_H_)
**A1**	H	−3.62	5.14	1.27	0.00	0.000
**A2**	Me	−3.48	5.03	1.21	−0.17	−0.019
**A3**	MeO	−3.29	4.76	1.13	−0.27	−0.046
**A4**	Br	−3.75	5.01	1.42	0.23	0.048
**A5**	F	−3.67	5.03	1.34	0.06	0.025
**A6**	Cl	−3.78	5.03	1.42	0.23	0.052
**A7**	COOH	−4.82	4.79	1.79	0.45	0.152
**A8**	COOMe	−4.05	4.82	1.71	0.45	0.130
**A9**	COMe	−4.16	4.65	1.86	0.50	0.169
**A10**	*tert-*butyl	−3.46	5.03	1.19	−0.20	−0.020
**A11**	CONH_2_	−3.97	4.93	1.60	0.36	0.103
**A12**	CN	−4.33	4.82	1.94	0.66	0.187
**B1**	H	−3.51	5.52	1.13	0.00	0.000
**B2**	Me	−3.37	5.39	1.06	−0.17	−0.028
**B3**	F	−3.54	5.47	1.14	0.06	0.003
**B4**	COOMe	−4.11	4.84	1.74	0.45	0.187
**B5**	PhO	−3.24	5.12	1.03	−0.03	−0.042
**B6**	CHO	−4.41	4.63	2.10	0.42	0.269
**B7**	Br	−3.73	5.22	1.33	0.23	0.070
**B8**	Cl	−3.75	5.28	1.32	0.23	0.067
**B9**	COOH	−4.22	4.87	1.82	0.45	0.206
**B10**	COMe	−4.22	4.71	1.90	0.50	0.225
**B11**	CN	−4.38	4.90	1.97	0.66	0.241
**B12**	tert-butyl	−3.35	6.07	0.92	−0.20	−0.089

^a^ Hammett substituent constants σ_p_ obtained from reference [20].

## Supplementary Materials

Supplementary materials can be accessed at: http://www.mdpi.com/1420-3049/21/11/1434/s1.
