# Supplementary material for: A Theoretical Study of the Relationship between the Electrophilicity ω Index and Hammett Constant σp in [3+2] Cycloaddition Reactions of Aryl Azide/Alkyne Derivatives
Source: Molecules. 2016 Oct 27;21(11):1434. doi: 10.3390/molecules21111434 (PMC6273986; doi:10.3390/molecules21111434)
Supplement: Supplementary file 1 [file molecules-21-01434-s001.pdf]

Supplementary Materials: A Theoretical Study of the Relationship between the Electrophilicity ω Index and Hammett Constant σp in [3+2] Cycloaddition Reactions of Aryl Azide/Alkyne Derivatives

Hicham Ben El Ayouchia, Hafid Anan, Moulay Lahcen El Idrissi Moubtassim, Luis R. Domingo, Miguel Julve and Salah-Eddine Stiriba

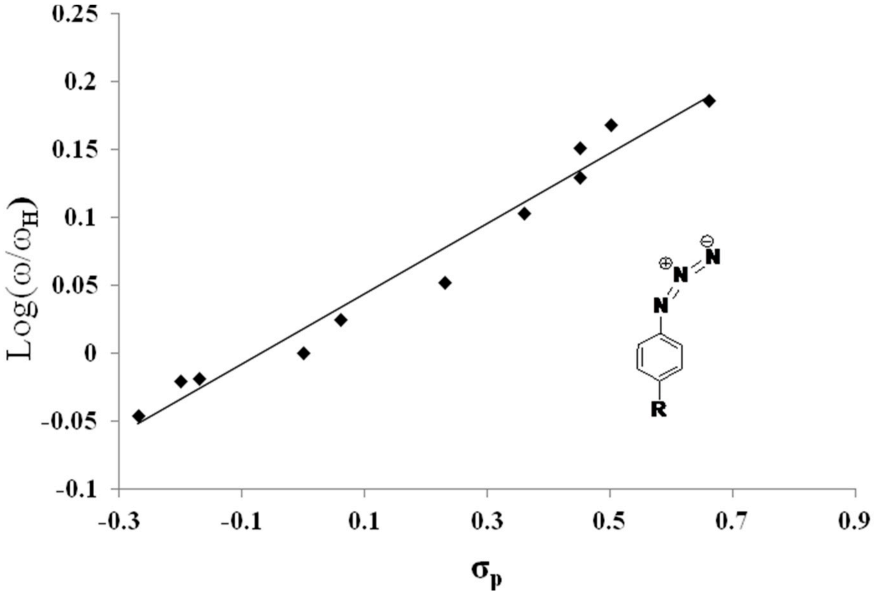

Figure S1. Plot of the logarithm of the global electrophilicity ratios of substituted azides versus the σ_p Hammett substituent constants. The value of the regression coefficient for the least- squares fit to a linear plot is $R^2 = 0.97$.

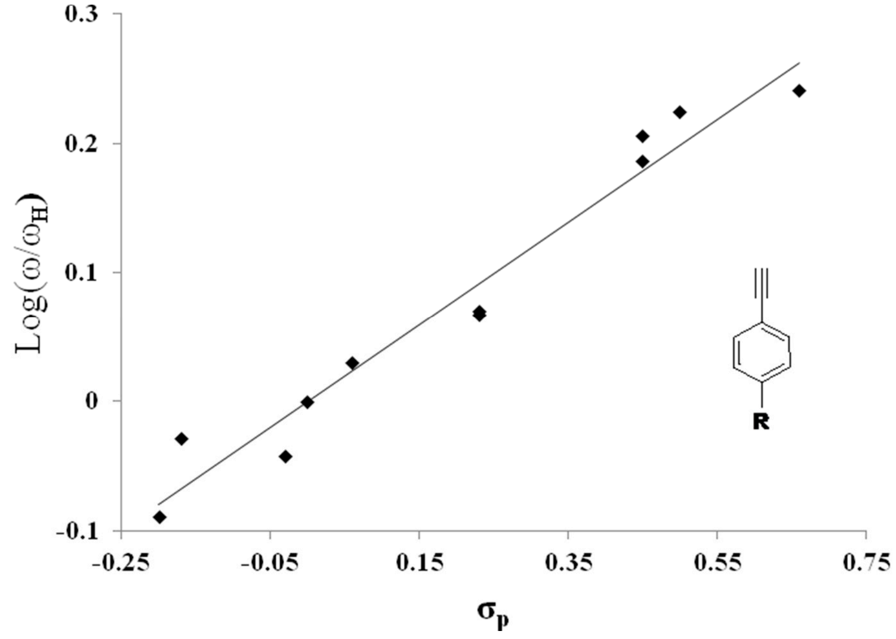

Figure S2. Plot of the logarithm of the global electrophilicity ratios of substituted alkynes versus the Hammett σ_p substituent constants. The value of the regression coefficient for the least- squares fit to a linear plot is $R^2 = 0.96$.
